# Development of Cellular and Enzymatic Bioluminescent Assay Systems to Study Low-Dose Effects of Thorium

**DOI:** 10.3390/bioengineering8120194

**Published:** 2021-11-29

**Authors:** Olga V. Kolesnik, Tatiana V. Rozhko, Maria A. Lapina, Vladislav S. Solovyev, Anna S. Sachkova, Nadezhda S. Kudryasheva

**Affiliations:** 1 Federal Research Center ‘Krasnoyarsk Science Center SB RAS’, Institute of Biophysics SB RAS, 660036 Krasnoyarsk, Russia; n-qdr@yandex.ru; 2 Biophysics Department, Siberian Federal University, 660041 Krasnoyarsk, Russia; strongerthan@mail.ru; 3 Krasnoyarsk State Medical Academy, 660022 Krasnoyarsk, Russia; gutniktv72@mail.ru; 4 National Research Tomsk Polytechnic University, 634050 Tomsk, Russia; vladislav.solovyev98@gmail.com (V.S.S.); asachkova@tpu.ru (A.S.S.)

**Keywords:** bioassay, bioluminescence, luminous bacteria, enzymes, reactive oxygen species, thorium, low-dose exposure, radiation hormesis

## Abstract

Thorium is one of the most widespread radioactive elements in natural ecosystems, along with uranium, it is the most important source of nuclear energy. However, the effects of thorium on living organisms have not been thoroughly studied. Marine luminescent bacteria and their enzymes are optimal bioassays for studying low-dose thorium exposures. Luminescent bioassays provide a quantitative measure of toxicity and are characterized by high rates, sensitivity, and simplicity. It is known that the metabolic activity of bacteria is associated with the production of reactive oxygen species (ROS). We studied the effects of thorium-232 (10^−11^–10^−3^ M) on *Photobacterium phosphoreum* and bacterial enzymatic reactions; kinetics of bacterial bioluminescence and ROS content were investigated in both systems. Bioluminescence activation was revealed under low-dose exposures (<0.1 Gy) and discussed in terms of “radiation hormesis”. The activation was accompanied by an intensification of the oxidation of a low-molecular reducer, NADH, during the enzymatic processes. Negative correlations were found between the intensity of bioluminescence and the content of ROS in bacteria and enzyme systems; an active role of ROS in the low-dose activation by thorium was discussed. The results contribute to radioecological potential of bioluminescence techniques adapted to study low-intensity radioactive exposures.

## 1. Introduction

Luminous marine bacterial bioassays are appropriate systems for radiotoxicity monitoring in complex multicomponent media. This bioassay has been utilized extensively [1,2,3,4,5] by utilizing bioluminescence intensity as a physiological testing parameter. The advantages of this bioassay include high sensitivity, simplicity, high-throughput capacity (1–3 min), and availability of devices for toxicity registration. These advantages provide the possibility of numerous sample analyses and proper statistical processing, with these being particularly important for investigating low-dose effects that are characterized as stochastic [6,7]. Bacterial bioluminescent assays can involve biological systems of different organization—bacterial or enzymatic.

Bacterial bioluminescent enzyme systems were suggested as a bioassay for the first time in 1990 [8] and the advantages of this approach were later demonstrated [2,3,4,9,10]. Primary physico-chemical processes in bioluminescent enzyme systems were reviewed [11,12]; mechanisms of exogenous compounds interactions with enzyme systems were discussed [13].

In our previous studies, we demonstrated the advantages of the bacteria-based and enzyme-based bioluminescent bioassays to monitor low-dose effects of radionuclide solutions [12,14,15,16,17,18,19,20,21] and gamma radiation [22]. It can be noted that in 2003 the luminous marine bacteria were applied to study the toxicity of radiation for the first time [23].

Low-intensity radioactive contaminations create problems in the vast territories of the world. According to ICRP *Publication 99* [24], a tentative limit of low doses for higher organisms is 0.1 Gy. In general, the toxicological literature suggests that the inhibition (suppression) of physiological functions of organisms separates harmful ‘radiotoxic’ intervals and ‘low-dose’ ranges with activating or threshold effects. This dose border can vary depending on the environmental conditions and organismal state [25,26].

Our previous works showed that the responses of luminous marine bacteria to the low-dose radiation of alpha and beta types [14,15,16,17,18,19,20,21,27,28] correspond to the conventional “hormesis” model [29,30,31,32]. This model includes, in the broadest case, three stages of the biological dose-dependent response—stress recognition, activation, and inhibition of organismal functions.

Molecular mechanisms of biological responses to low-intensity radiation are of practical interest; they allow for the prediction of the response of living organisms to low-intensity radiation in large territories after nuclear accidents, discharges of nuclear plants, or underground mining.

The molecular mechanisms of the radionuclide bioeffects are conventionally attributed to reactive oxygen species (ROS) that are generated as products of radioactive decay in water bodies in the presence of dissolved molecular oxygen [18,33,34,35]. It is known that ROS might be moderately consumed by water microorganisms [36,37]. On the other hand, ROS are native products of metabolic oxidative processes in living organisms [38,39,40]. Our previous results [6,16,17] have demonstrated that luminous marine bacteria naturally increase ROS content in aquatic media and intensify the ROS production in the presence of the radionuclide tritium.

Chemically, ROS are products of partial reduction of oxygen; the ROS family includes hydroxyl radicals (OH^•^), hydrogen peroxide (H_2_O_2_), superoxide anion (O_2_^•−^), and others [39].

According to modern approaches, ROS are involved in damage and signaling pathways [41,42]; they regulate organismal functions, such as apoptosis or protective responses in cells [33]. ROS are responsible for proliferation, migration, differentiation, and metabolism [43,44]; they are considered as stimulators of a cell division [45,46] or cell death—apoptosis, autophagy, and necrosis [47,48]. The signaling function of ROS is intensively discussed now [49,50,51]; a pioneer in this approach is Proctor [45]. It should be noted that ROS can serve both as inter- or intra-cellular messengers [52,53,54]. Reactive oxygen or nitrogen [55] species released by cells can serve as signals that elicit the radiation-induced ‘bystander effect’ [56,57].

Previously [6,16,17], we found correlations between the ROS content and bacterial bioluminescence intensity in tritiated water and thus suggested the involvement of ROS in the activation of bacterial physiological functions under exposure to the beta-emitting radionuclide tritium. A question of special interest is: are ROS effects responsible for the bioeffects of other radionuclides with differing characteristics of radioactive decay?

Alpha emitting radionuclide thorium (Th) is of particular interest as it is one of the most abundant radioactive elements in natural ecosystems. Its longest-lived isotope, Th-232, has a half-life of 14 × 10^9^ years. In the environment, thorium occurs predominantly in a tetravalent state and is present in trace amounts in phosphates, simple and multiple oxides, and silicates [58]. It is found in coal, which is used as fuel for urban thermal power plants [59,60]. Along with uranium, thorium is considered as the most important source of nuclear energy and is currently receiving attention due to its application potential as a cleaner, safer, and more widespread nuclear fuel. Additionally, thorium is used for production of ceramics, welding rods, lenses for cameras and telescopes, refractory bricks, heat-resistant paints, and materials for the aerospace industry.

On the other hand, since thorium is naturally present in air, water, soil and biological materials, humans are perpetually exposed to small amounts of thorium that are ingested through inhalation, ingestion, and skin penetration. There exist large areas contaminated with thorium, which may exert detrimental effects for decades [61]. This challenge motivates modern studies of thorium toxicity at the cellular and molecular levels [62,63,64]. However, at present, the effect of radioactive thorium on living organisms has not been sufficiently studied.

The aim of the current work is to study the effects of thorium on bioluminescent bioassays at the cellular, biochemical, and molecular levels; low-concentration effects of thorium are under special consideration. Our paper considers the responses of (1) luminous marine bacteria, (2) coupled system of enzymatic reactions of marine bacteria, and (3) the low-molecular component of the enzymatic system, endogenous reducer NADH, to Th-232 exposure. Monitoring of ROS content under the exposure conditions is provided; correlations between the ROS content and the responses of the bioluminescence assay systems to Th-232 are analyzed to highlight ROS involvement in the bacterial cell response. The results contribute to the adaptation of bioluminescence bioassays, cellular and enzymatic, for monitoring low-intensity radioactive exposures under natural conditions.

## 2. Materials and Methods

### 2.1. Preparations and Reagents

Intact marine luminous bacterium, strain *Photobacterium phosphoreum* 1883 IBSO [65], was used to evaluate the effects of Th-232 on the cellular system. The strain was obtained from the Collection of Luminous Bacteria CCIBSO-863, Institute of Biophysics SB RAS, Krasnoyarsk, Russia. For the cultivation of *P. phosphoreum*, the semisynthetic medium containing: 10 g/L Tryptone, 28.5 g/L NaCl, 4.5 g/L MgCl_2_∙6H_2_O, 0.5 g/L CaCl_2_, 0.5 g/L KCl, 3 g/L yeast extract, and 12.5 g/L Agar was used. The NaCl was of analytical grade, it was applied to prepare bacterial suspensions for the bioluminescence measurements. The 3% NaCl solutions were used to imitate a marine environment for the bacterial cells and to balance osmotic processes.

The enzymatic bioassay was based on the kit, which included lyophilized preparations of luciferase (0.5 mg/mL) and NADH:FMN-oxidoreductase (0.15 units of activity). The kit was produced at the Institute of Biophysics SB RAS, Krasnoyarsk, Russia. The chemicals for the enzymatic assay were: NADH from ICN, USA; FMN and tetradecanal from SERVA, Germany.

Reagents for the chemiluminescence measurements were: luminol from Sigma-Aldrich; hydrogen peroxide solution, H_2_O_2_, from Tula Pharmaceutical Factory, Russia; K_3_[Fe(CN)_6_] from Khimreaktiv, Russia. All reagents were of chemical grade.

Preparation of Th-232, Th(NO_3_)_4_∙4H_2_O, was used to prepare radioactive solutions. The solutions contained: Th-232 > 98.6%, sulfates SO_4_^2−^—0.005%, chlorides Cl^−^—0.002%, iron (Fe), cerium (Ce)—0.05%, phosphorus oxide (P_2_O_5_)—0.005%, Al, Ca, Mg—0.05%. Specific radioactivity of Th-232—1.27 × 10^5^ Bq/kg.

### 2.2. Bioluminescence Assay System Composition

#### 2.2.1. Bacterial Assay

The bacterial suspension samples were prepared according to a standard technique; the bacterial suspensions were prepared as follows: Control non-radioactive samples: 20 μL of bacterial suspensions were added to 160 μL of 3% NaCl solution. Radioactive samples: 20 μL of bacterial suspensions and 18 μL of Th-232 (10^−10^–10^−2^ M) were added to 142 μL of 3% NaCl solution.

#### 2.2.2. Enzymatic Assay

The bioluminescent system of two coupled reactions catalyzed by the NAD(P)H:FMN-oxidoreductase and luciferase was used to monitor effects of thorium in the solutions.

The solutions of chemicals were prepared as follows: NADH was dissolved in 0.05 M potassium phosphate buffer, pH 6.8. To prepare a solution of 0.0025% tetradecanal (RCHO), 5 mL of 0.05 M potassium phosphate buffer (pH 6.8) was added to 50 μL of a 0.25% alcohol solution of aldehyde. The enzyme preparation kit was dissolved in 2 mL of 0.05 M potassium phosphate buffer.

The control (non-radioactive) samples were of the following composition:

- 6 μL solution of the enzyme preparation;
- 25 μL 5.4 × 10^−4^ M FMN;
- 25 μL 0.0025% RCHO;
- 50 μL 0.05 M potassium phosphate buffer (pH 6.8);
- 100 μL 4 × 10^−4^ M NADH.

Radioactive samples were of the following composition:

- 6 μL solution of the enzyme preparation in a Th-232 (3.5 × 10^−2^ M–3.5 × 10^−8^ M) solution;
- 25 μL 5.4 × 10^−4^ M FMN;
- 25 μL 0.0025% RCHO;
- 50 μL 0.05 M potassium phosphate buffer (pH 6.8);
- 100 μL 4 × 10^−4^ M NADH.

#### 2.2.3. Effect of Th-232 on NADH Oxidation Rates

Effects of Th-232 on NADH oxidation rates were studied using the solutions of different compositions:

1. NADH: 200 μL 4 × 10^−4^ M NADH; 255 μL 0.05 M potassium phosphate buffer (pH 6.8); 55 μL distilled H_2_O;
2. NADH + enzyme preparation: 200 μL 4 × 10^−4^ M NADH; 5 μL enzyme preparation; 250 μL 0.05 M potassium phosphate buffer (pH 6.8); 55 μL distilled H_2_O;
3. NADH + FMN: 200 μL 4 × 10^−4^ M NADH; 50 μL 5 × 10^−4^ M FMN; 255 μL 0.05 M potassium phosphate buffer (pH 6.8); 5 μL distilled H_2_O;
4. NADH + FMN + enzyme preparation: 200 μL 4 × 10^−4^ M NADH; 5 μL enzyme preparation; 250 μL 0.05 M potassium phosphate buffer (pH 6.8); 50 μL 5 × 10^−4^ M FMN; 5 μL distilled H_2_O;
5. NADH + Th-232: 200 μL 4 × 10^−4^ M NADH; 50 μL 10^−6^ M Th-232; 205 μL 0.05 M potassium phosphate buffer (pH 6.8); 55 μL distilled H_2_O;
6. NADH + enzyme preparation + Th-232: 200 μL 4 × 10^−4^ M NADH; 5 μL enzyme preparation; 50 μL 10^−6^ M Th-232; 200 μL 0.05 M potassium phosphate buffer (pH 6.8); 55 μL distilled H_2_O;
7. NADH + FMN + Th-232: 200 μL 4 × 10^−4^ M NADH; 50 μL 10^−6^ M Th-232; 50 μL 5 × 10^−4^ M FMN; 205 μL 0.05 M potassium phosphate buffer (pH 6.8); 5 μL distilled H_2_O;
8. NADH + FMN + enzyme preparation + Th-232: 200 μL 4 × 10^−4^ M NADH; 5 μL enzyme preparation; 50 μL 10^−6^ M Th-232; 200 μL 0.05 M potassium phosphate buffer (pH 6.8); 50 μL 5 × 10^−4^ M FMN; 5 μL distilled H_2_O.

### 2.3. Bioluminescence Registration

To investigate the chronic effects of the low-level radiation of thorium on the bioluminescence of cellular and enzyme systems, standard procedures for the bioluminescence measurements were used [6,65]. All bioluminescence measurements were carried out in four replicates. Bioluminescence intensities were measured during 32 h and 150 min for bacterial and enzymatic assays, respectively. The relative bioluminescence intensities, *I^rel^* (relative units, r.u.), were calculated as ratios of the bioluminescence intensities in radioactive suspensions to these in non-radioactive suspensions. Time-courses of *I^rel^* were studied at different concentrations of Th-232 (10^−11^–10^−3^ M). The dose accumulated in the bacterial culture did not exceed 0.1 Gy.

### 2.4. Chemiluminescence Measurements

The luminol chemiluminescence method [66,67] was used. This method determines an integral content of ROS assuming that a dynamic equilibrium of the different ROS forms takes place. Additionally, the chemiluminescence-based technique is highly convenient in a complex application with bioluminescence measurements, as these two techniques use the same instrumentation for the light-emitting registration. The chemiluminescence registration was carried out immediately following the bioluminescence measurements in the same bacterial samples. 

The calibration dependence was preliminarily determined as a chemiluminescence intensity vs. H_2_O_2_ concentration; H_2_O_2_ was applied here as a ROS model. Concentrations of alkaline luminol and K_3_[Fe(CN)_6_] solutions were 5 × 10^−4^ M and 10^−3^ M, respectively. The calibration dependence is presented in Supplementary Materials Figure S1.

Chemiluminescence intensity was evaluated at different Th-232 concentrations (10^−11^–10^−3^ M) in 3% NaCl solutions at various times of exposure. The alkaline luminol solutions, pH 11.24, were added to the bacterial/enzyme samples. Then, the chemiluminescence reaction was initiated by 75 μL solution of K_3_[Fe(CN)_6_] through the injection system. Chemiluminescence intensity was measured and used to calculate ROS content in the experimental solutions via the calibration dependence (Supplementary Materials Figure S1). Relative values of ROS content (*ROS^rel^*, r.u.) were calculated as ratios of ROS content in the radioactive solutions to that in the non-radioactive solutions; kinetics of *ROS^rel^* was plotted.

### 2.5. Acidity Measurements

The pH of thorium solutions (10^−11^–10^−3^ M) was measured in the presence of luminol (5 × 10^−4^ M). The pH of luminol solutions was 11. The measurements were carried out in 3 replications; error did not exceed 2%.

### 2.6. Equipment

Luminescence intensity was registered by Luminoskan Ascent (Thermo Fisher Corp., Waltham, MA, USA). Optical density, *D*, of the solutions was measured by a double-beam spectrophotometer UVIKON-943 (KONTRON Instruments, Milano, Italy). All measurements were carried out at +20 °C.

### 2.7. Statistical Processing

To evaluate correlations between bioluminescence signal and ROS concentrations, a statistical dependence between the rankings of two variables was analyzed [68] and Spearman’s rank correlation coefficients *r* were calculated. The application of this method was justified with a moderate kit of data set, as well as a lack of normal distribution of bioluminescence intensity and ROS content.

## 3. Results

### 3.1. Effect of Thorium on Bioluminescence of Bacteria

**Figure 1:**
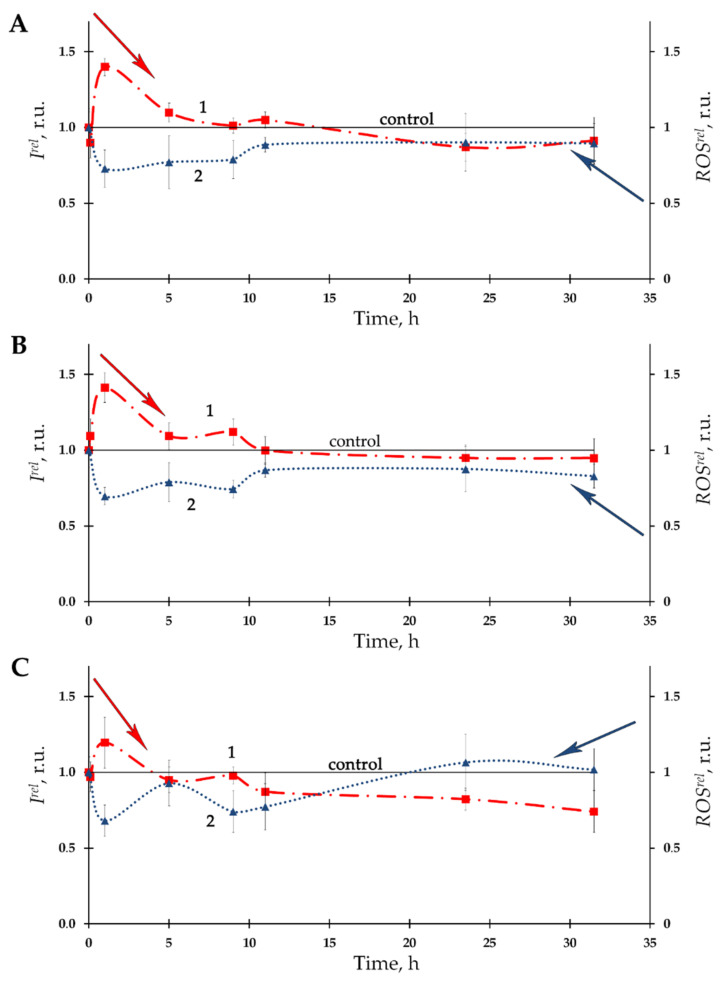
Kinetics of bacterial bioluminescence, *I^rel^*, (1) and relative ROS content, *ROS^rel^*, (2) in the presence of Th-232 of different concentrations: (**A**)—10^−11^ M; (**B**)—10^−10^ M; (**C**)—10^−7^ M. The ROS content in the control (non-radioactive) bacterial suspension decayed from 5.9 × 10^−6^ M to 1.7 × 10^−6^ M for the time of experiment.

Dependences of bacterial bioluminescence intensity on time of exposure to Th-232 were studied at different concentrations of the radionuclide solutions (10^−11^–10^−3^ M). Figure 1A–C presents the examples of the bioluminescence kinetic curves (curves 1) at three thorium concentrations—10^−11^, 10^−10^, and 10^−7^ M, which were chosen as examples. It is seen that all three dependencies involve moderate bioluminescence activation (*I^rel^* >1) during the initial stage of exposure as well as an absence of effects during the last stage of the exposure.

Activation of bacterial bioluminescence was previously observed in diluted solutions of another alpha-emitting radionuclide, americium-241, with higher energy of radioactive decay [14,18,20,21]; the effect was attributed to radiation hormesis.

The time-course of ROS content in the bacterial suspension is presented at Figure 1A–C as curves 2. A moderate decrease of ROS content (as compared to nonradioactive control) with a tendency towards restoration was observed. The negative correlations between *I^rel^* and *ROS^rel^* (A: *r* = –0.85; B: *r* = –0.67: C: *r* = –0.89, *p* < 0.05) were found, thus demonstrating inverse interrelations between bacterial bioluminescence intensity and ROS concentration in the bacterial environment.

**Figure 2:**
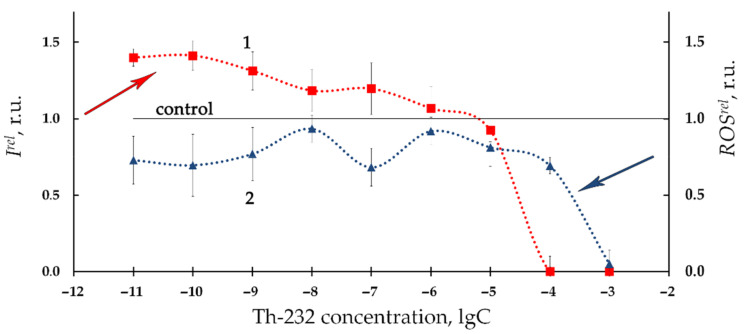
Relative bioluminescence intensity, *I^rel^*, (1) and relative ROS content, *ROS^rel^*, (2) in bacterial suspension at different concentrations of Th-232, M. Time of exposure to Th-232 was 1 h. ROS content in the control (non-radioactive) sample was 5.5 × 10^−6^ M.

Figure 2 presents the relative bioluminescence intensity, *I^rel^,* (curve 1) and relative ROS content, *ROS^rel^*, (curve 2) in bacterial suspension at different concentrations of Th-232 (10^−11^–10^−3^ M). Low-concentration (10^−11^–10^−6^ M) activation of bioluminescence is evident from Figure 2 (*I^rel^* >1). In addition, Figure 2 demonstrates bacterial bioluminescence inhibition (*I^rel^* < 1) at higher concentrations of thorium solutions (10^−6^–10^−3^ M).

Additionally, Figure 2 demonstrates the lower ROS content as compared to nonradioactive (control) solutions (*ROS^rel^* < 1, curve 2) in a wide concentration interval of Th-232. The most pronounced decay of ROS was observed at higher thorium concentrations (>10^−5^ M), similar to the bioluminescence intensity fall (*I^rel^* < 1, curve 1, Figure 2).

We analyzed correlations between *I^rel^* and *ROS^rel^* in the bacterial suspensions at low-concentration range of thorium (10^−11^–10^−6^ M, Figure 2). This range revealed a negative correlation (r = −0.60, *p* < 0.05). This result demonstrates inverse correlations between bioluminescence intensity and ROS content. We can conclude that the low-concentration bacterial bioluminescence activation is concerned with intensification of redox processes in the water media and consumption of ROS by the bacteria [36,37].

The high concentration interval of thorium (10^−6^–10^−3^ M, Figure 2) did not reveal negative correlations. This absence of correlations is likely indicative of complex mechanisms involved in bacterial bioluminescence inhibition. One of the additional mechanisms might be concerned with the high acidity of the high-concentration Th(NO_3_)_4_ solutions. It is known [69] that a sharp decline in bioluminescence activity of *Ph. phosporeum* takes place at pH 5 and lower. The pH-values of the bacterial suspensions were found in our experiments as 5.4, 4.9, 4.0, and 3.4 at thorium concentrations 10^−6^ M, 10^−5^ M, 10^−4^ M, and 10^−3^ M, respectively. Hence, the high-concentration decay of bioluminescence intensity (*I^rel^* < 1, curve 1, Figure 2) can be explained by increased acidity in the bacterial suspension following the addition of acidic solutions of Th(NO_3_)_4_.

**Figure 3:**
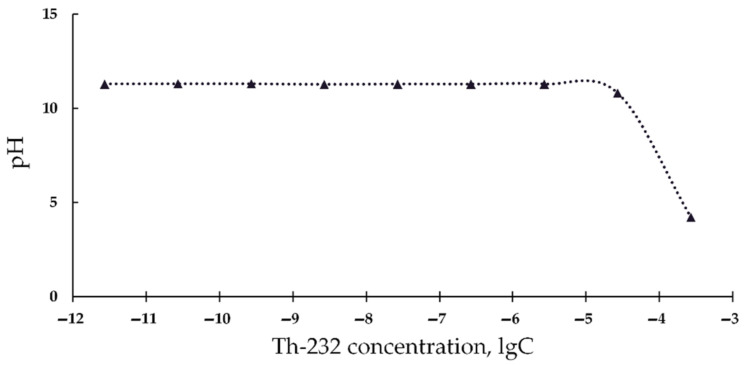
pH values of luminol solutions vs. concentration of Th(NO_3_)_4_, M.

Additionally, it is known that the chemiluminescence luminol method for ROS monitoring uses pH 11 [66,67], hence, it cannot be applied in acidic solutions. To verify this assumption we measured the acidity of the alkaline luminol solutions under addition of acidic Th(NO_3_)_4_ solutions (Figure 3). It is seen that high Th-232 concentrations (>10^−6^ M) decrease the pH of the solutions. This suggests the unsuitability of the chemiluminescence luminol method for ROS monitoring at high concentrations of our thorium preparation.

Therefore, the high concentration Th(NO_3_)_4_ solutions (>10^−6^ M) should be excluded from the analysis of the physico-chemical ROS-dependent processes in radioactive bacterial suspensions.

We can conclude that the low-concentration bioluminescence activation by Th-232 (10^−11^–10^−6^ M) is concerned with the decrease of ROS in the bacterial suspension. This conclusion infers the mechanism of “hormetic” response of the bacterial cells to low-intensity radiation of Th-232.

Our previous study [6] also demonstrated bacterial bioluminescence activation under low-dose radioactive exposures to beta-emitting radionuclide tritium, however, the activation was found to be more effective at the tritium exposure (up to 300%) than in our current study using thorium exposure (not greater than 50%, Figure 1 and Figure 2 curves 1). Opposite correlations with ROS were observed in the aforementioned paper: 300% increase of ROS content was associated with bacterial bioluminescence activation by tritium, while our current study demonstrates a moderate ROS decrease at low-concentration exposure to thorium (Figure 1 and Figure 2 curves 2). Such opposite tendencies in ROS change in bacterial suspensions may be concerned with the atomic weights of the isotopes of tritium and thorium, as well as the different energies of their radioactive decay.

It was demonstrated earlier that heavier atoms inhibit bacterial bioluminescence to a higher degree as compared to lighter atoms [70,71,72]. This pattern was concerned [70] with atomic electronegativity, which contributed to efficient electron acceptance by heavy metals, with this providing a basic physicochemical explanation for their multiple effects on complex living systems: interactions with enzymes and substrates, disturbing the free-radical balance in cells and intracellular media, involving ROS balance, and so on. In [73], the inhibiting and activating effects of heavy isotopes, stable and radioactive (uranium-235 + 238, americium-241, and europium) were discussed, taking into consideration their electron acceptance ability and radioactivity. Thorium-232 and uranium-238, being the most abundant radioactive heavy elements in nature, are similar in radioactive decay energy (4.08 and 5.63 MeV, respectively), and comparable in specific radioactivity (4 and 12 kBq, respectively) [74], however, salts of thorium are of lower toxicity as compared with uranyl salts [75].

The differences require further consideration, and the results presented below use enzymatic reactions to elucidate the underlying processes.

### 3.2. Effect of Th-232 on Bioluminescence of Enzymatic Assay

**Figure 4:**
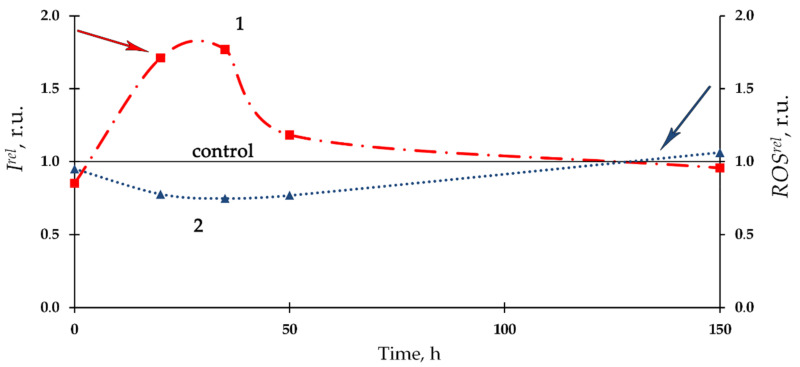
Kinetics of the relative bioluminescence intensity, *I^rel^*, (1) and relative ROS content, *ROS^rel^* (2) in the bacterial enzyme system in the presence of Th-232, C = 10^−7^ M. The ROS content in the control (non-radioactive) solutions increased from 0.9 × 10^−7^ M to 6.6 × 10^−7^ M for the time of experiment.

Figure 4 presents the dependence of relative bioluminescence intensity, *I^rel^,* and ROS content, *ROS^rel^*, in the bacterial enzyme system on time of exposure to Th-232. The 10^−7^ M thorium solution was chosen as an example. Bioluminescence activation (*I^rel^* > 1, curve 1) and a slight decrease of ROS content (*ROS^rel^ <* 1, curve 2) were observed at the initial stage of the bioluminescence process, with mitigation of the deviations from the control during the later stage. Negative correlation (*r* = −0.80) between *I^rel^* and *ROS^rel^* was revealed, showing similarities with the bacterial cellular system (Section 3.1, Figure 1).

Notably, we observed an increase in ROS content in the control (nonradioactive) solutions of the enzyme system (as stated in the caption for Figure 4), in contrast to the bacterial system (caption for Figure 1). Probably, this increase can be explained with dark processes associated with the accumulation of peroxide compounds in the reaction of bacterial luciferase [76]. Bacterial cells are likely able to utilize the excess of ROS produced in the enzymatic reactions if functioning under the conditions close to their metabolic balance.

**Figure 5:**
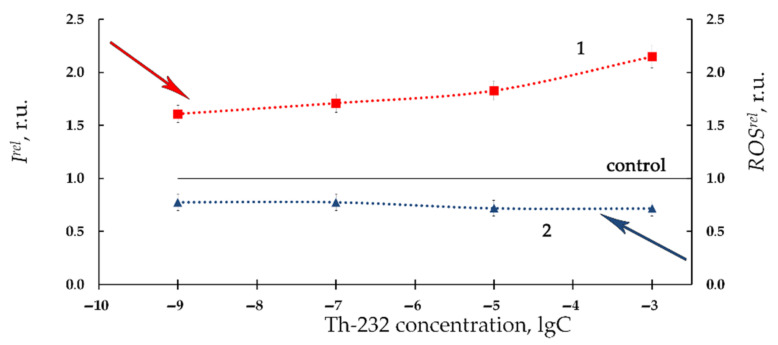
Relative bioluminescence intensity, *I^rel^*, (1) and relative ROS content, *ROS^rel^*, (2) in the enzyme system at different concentrations of Th-232, M. Time of exposure to Th-232—20 min. ROS content in the control (non-radioactive) sample—1.4 × 10^−7^ M.

Figure 5 presents the dependencies of *I^re^* and *ROS^rel^* on concentrations of Th(NO_3_)_4_ in the enzyme system at 20 min exposure (curves 1 and 2, respectively). Bioluminescence activation (*I^rel^* > 1, Figure 5, curve 1) was observed in the enzyme solutions, similar to the bacterial suspension, Figure 2, curve 1.

However, in contrast to the bacterial system, higher concentrations of Th-232 did not inhibit the enzymatic bioluminescence (compare Figure 2 and Figure 5, curves 1). A moderate decrease of ROS content (*ROS^rel^* < 1) was observed at all concentrations of Th(NO_3_)_4_ (Figure 5, curve 2). We did not observe a considerable ROS decrease at higher thorium concentrations, opposite to the bacterial suspensions (Figure 2 and Figure 5, curves 2). We suppose that these higher-concentration differences result from the different acidity in the bacterial suppressions (pH < 5.4, as discussed before in Section 3.1) and buffer solutions used for the enzymatic assay (pH 6.8).

A strong negative correlation (r = −0.99, *p* < 0.05) between the concentration dependencies of *I^rel^* and *ROS^rel^* was found during the time of exposure to Th-232, Figure 5. The interrelation of the enzymatic bioluminescence activation with ROS decrease refers to the mechanism of the bacterial bioluminescence reaction: it is known that the intermediates of the reaction, flavin peroxy-hemiacetal [11,76,77], is a peroxide, i.e., a representative of ROS. Hence, the moderate decrease in ROS content (*ROS^rel^* < 1, Figure 5) in Th-232 solutions can be a consequence of the intensification of the bioluminescent reaction, and hence, activation of light emission.

This intensification of ROS consumption in the presence of Th-232 should result in increased rates of redox processes, enzymatic and non-enzymatic, particularly the rates of NADH oxidation. We assessed this supposition; the results are presented below.

### 3.3. Effect of Th-232 on NADH Oxidation Rates

We studied the rates of NADH oxidation (V) in solutions of different compositions involving components of the bioluminescent enzymatic systems: FMN and enzyme preparation. The rates were determined in the absence and presence of Th(NO_3_)_4_, C = 10^−7^ M, Table 1.

The increase of NADH oxidation rates under exposure to Th-232 is evident in all solutions (1–4, Table 1). The increase in rates was within 1.5–1.7. This result indicates that thorium increases the efficiency of redox processes involving biologically important molecules (NADH and FMN) and enzymes. This intensification may be related to the decrease of ROS content in the enzyme solutions as compared to control (*ROS^rel^* < 1, Figure 5, curve 2).

## 4. Conclusions

Our current study has demonstrated the bioeffects of thorium, one of the most widespread radioactive elements on Earth; its low-intensity influence on living systems justifies its investigation for the prognoses of organismal states in environments of different radioactive characteristics. We used simplified assay systems with bioluminescence indicators—cellular (luminous marine bacteria) and enzymatic (system of coupled enzyme reactions), which we supposed to be the most convenient bioassays among the existing ones, adapted for studying of low-intensity radioactive exposures.

We found that Th-232 moderately activates cellular and enzymatic processes under low-dose exposures (<0.1 Gy). The activation processes were accompanied by a consumption of reactive oxygen species and an intensification of the oxidation of the low-molecular reducer, NADH, in the enzymatic processes. The results contribute to understanding the molecular mechanism of “hormetic” responses of cells to low-intensity radioactive exposures; Th-232 was used here as a representative of a group of alpha-emitting radionuclides.

The results contribute to understanding the potential of bioluminescence bioassays for monitoring the low-intensity radioactive exposures and further adaptation of bioluminescent techniques to radioecological monitoring.

## Figures and Tables

**Table 1:** Rates of NADH oxidation (V) in solutions of different composition. Wavelength of OD registration was 340 nm. Concentration of Th(NO_3_)_4,_ was 10^−7^ M.

Number of Solutions	Components of Solutions	V × 10^8^, M	
		without Th	with Th
1	NADH	2.43	4.05
2	NADH + enzyme preparation	4.05	6.07
3	NADH + FMN	14.20	20.60
4	NADH + FMN + enzyme preparation	16.20	26.70

## Supplementary Materials

The following are available online at https://www.mdpi.com/article/10.3390/bioengineering8120194/s1, Figure S1: Calibration curve for chemiluminescence luminol method for ROS evaluation. Dependence of chemiluminescence intensity on concentration of H_2_O_2_.
