# Supplementary material for: Development of Cellular and Enzymatic Bioluminescent Assay Systems to Study Low-Dose Effects of Thorium
Source: Bioengineering (Basel). 2021 Nov 29;8(12):194. doi: 10.3390/bioengineering8120194 (PMC8698266; doi:10.3390/bioengineering8120194)
Supplement: Supplementary file 1 [file bioengineering-08-00194-s001.zip › bioengineering-1459656-supplementary.pdf]

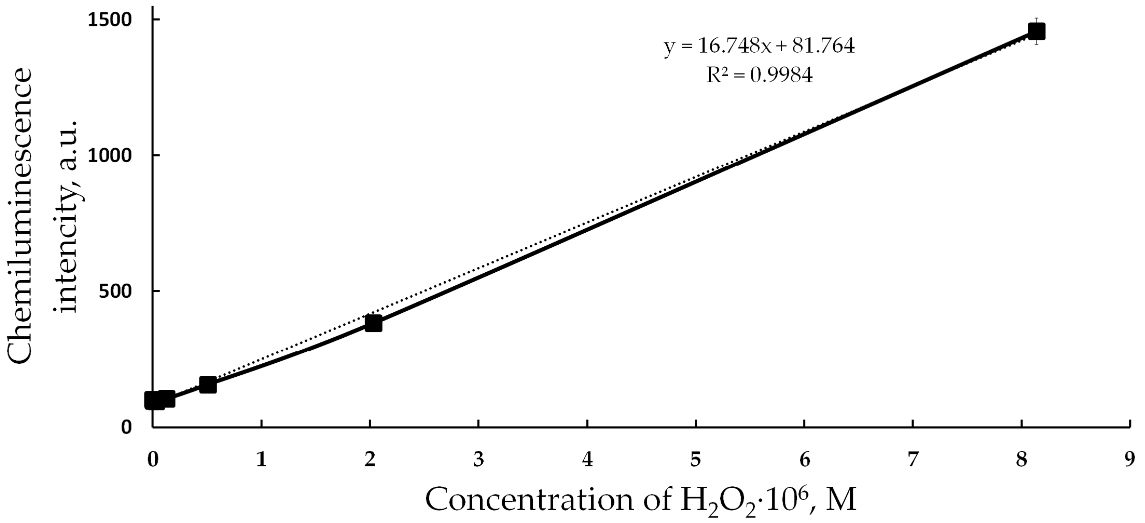

Figure S1. Calibration curve for chemiluminescence luminol method for ROS evaluation. Dependence of chemiluminescence intensity on concentration of H_2O_2.
